# Association of *HADHA *expression with the risk of breast cancer: targeted subset analysis and meta-analysis of microarray data

**DOI:** 10.1186/1756-0500-5-25

**Published:** 2012-01-12

**Authors:** Manju Mamtani, Hemant Kulkarni

**Affiliations:** 1 Lata Medical Research Foundation, Nagpur, India; 2 Texas Biomedical Research Institute, 7620 NW Loop 410, San Antonio, Texas 78227-5301, USA

**Keywords:** Breast cancer, *HADHA *gene, Gene expression profiling, Microarray, Meta-analysis

## Abstract

**Background:**

The role of n-3 fatty acids in prevention of breast cancer is well recognized, but the underlying molecular mechanisms are still unclear. In view of the growing need for early detection of breast cancer, Graham et al. (2010) studied the microarray gene expression in histologically normal epithelium of subjects with or without breast cancer. We conducted a secondary analysis of this dataset with a focus on the genes (n = 47) involved in fat and lipid metabolism. We used stepwise multivariate logistic regression analyses, volcano plots and false discovery rates for association analyses. We also conducted meta-analyses of other microarray studies using random effects models for three outcomes--risk of breast cancer (380 breast cancer patients and 240 normal subjects), risk of metastasis (430 metastatic compared to 1104 non-metastatic breast cancers) and risk of recurrence (484 recurring versus 890 non-recurring breast cancers).

**Results:**

The *HADHA *gene [hydroxyacyl-CoA dehydrogenase/3-ketoacyl-CoA thiolase/enoyl-CoA hydratase (trifunctional protein), alpha subunit] was significantly under-expressed in breast cancer; more so in those with estrogen receptor-negative status. Our meta-analysis showed an 18.4%-26% reduction in *HADHA *expression in breast cancer. Also, there was an inconclusive but consistent under-expression of *HADHA *in subjects with metastatic and recurring breast cancers.

**Conclusions:**

Involvement of mitochondria and the mitochondrial trifunctional protein (encoded by *HADHA *gene) in breast carcinogenesis is known. Our results lend additional support to the possibility of this involvement. Further, our results suggest that targeted subset analysis of large genome-based datasets can provide interesting association signals.

## Background

Early detection of malignant breast neoplasms is critical to cancer prevention and treatment. Cancer chemoprevention (also called as treatment of carcinogenesis) is a primordial prevention step that is receiving considerable attention. In that context, the identification of an ideal biomarker for breast cancer has become increasingly important. In spite of the vast number of studies conducted in the past; a recent, comprehensive and elegant review argues that there is still a lack of clarity regarding the understanding of the process of breast carcinogenesis [1]. Interestingly, it has been demonstrated that the mammary gland basal cells have features consistent with the progenitor stem cells and that they can differentiate into benign or malignant lesions intraductally [2]. It has also been shown in murine models that differentiated intact mammary glands can exert a negative influence on the development of breast cancer [3]. However, the search for an ideal breast cancer biomarker is still on [4].

A logical undertaking in this direction is the use of microarrays to study the differential gene expressions in breast cancer. Consistent with the spirit of research that encourages very early detection of carcinogenesis, Graham et al. [5] recently studied histologically normal epithelium from subjects with and without breast cancer and identified an 86-gene signature that indicated a genomic change prior to carcinogenesis. They found that many of these genes belonged to the family of growth factors, cytokines, oxidative stress modifiers, p38 MAP kinase pathway members, transcription regulators or determinants of nucleic acid stability [5].

Interestingly they did not find genes associated with fatty acid or lipid metabolism to be differentially expressed in histologically normal epithelium. Derangements of fatty acid and lipid metabolism have been implicated in oncogenesis in many studies, especially in the cancer of breast [6]. It is generally believed that diets rich in n-6 polyunsaturated fatty acids (n-6 PUFA) and saturated fatty acids (SFA) increase the risk of tumorigenesis while diets rich in n-3 polyunsaturated fatty acids (n-3 PUFA) reduce the risk of cancer development [7-10]. Lipids have the ability to influence the process of neoplasia via their effects on hormone status, cell membrane integrity, signal transduction, immune modulation and regulation of gene expression [11,12]. In this study, we specifically examined whether the genes related to fatty acid and lipid metabolism are also differentially associated with breast cancer status. For this, we used a targeted subset analysis of the microarray data from Graham et al. [5] and also conducted meta-analyses of other microarray datasets.

## Methods

### The primary dataset

The microarray dataset used in the present study is available for public use on the Gene Expression Omnibus website http://www.ncbi.nlm.nih.gov/sites/GDSbrowser?acc=GDS3716 of the National Institutes of Health, USA. Details of the study subjects on whom these microarray studies were conducted have been described previously [5]. Briefly, the dataset comprises microarray data collected through Affymetrix Human Genome U133A platform that measures expression of 22,283 genes. The data were collected using histologically normal epithelium from four sets of subjects--those who underwent reduction mammoplasty (n = 18), those who underwent preventive mastectomy (n = 6), estrogen receptor positive (ER+) breast cancer patients (n = 9) and estrogen receptor negative (ER-) breast cancer patients (n = 9). The data were available in normalized format.

### Targeted subset analysis

Our main aim was to assess if genes related with fatty acid and lipid metabolism were differentially expressed in the study dataset. For this we first culled a list of genes that have been implicated in the fatty acid and lipid metabolism. We used the DAVID http://david.abcc.ncifcrf.gov and KEGG Pathway http://www.genome.jp/kegg/metabolism.html websites and generated a list of 136 genes implicated in one or more of the following pathways: fatty acid metabolism, fatty acid biosynthesis, peroxisome proliferator-activated receptor (PPAR) signaling pathway, lipopolysaccharide biosynthesis, lipid metabolism and fat digestion and absorption. A full list with functional annotation of these 136 genes is provided as Additional file 1: Table S1. We then used the DAVID http://david.abcc.ncifcrf.gov and Clone/Gene ID Converter http://idconverter.bioinfo.cnio.es/IDconverter.php programs to find out which of these 136 genes were included in the Affymetrix Human Genome U133A platform. We found 47 probe sets related to genes (Table 1) that partake in lipid or fatty acid metabolism to be represented in the study datasets. We conducted our analyses on the potential differential expression of these 47 genes. Complete functional annotation for these 47 genes is provided in Additional file 2: Table S2.

**Table 1:** Genes included in the analyses

#	Symbol	Affymetrix Probe Set Id	Gene Name
1	*ACAA1*	202025_x_at	acetyl-Coenzyme A acyltransferase 1

2	*ACADL*	206068_s_at	acyl-Coenzyme A dehydrogenase, long chain

3	*ACADM*	202502_at	acyl-Coenzyme A dehydrogenase, C-4 to C-12 straight chain

4	*ACAT1*	205412_at	acetyl-Coenzyme A acetyltransferase 1

5	*ACSBG2*	221716_s_at	acyl-CoA synthetase bubblegum family member 2

6	*ACSL3*	201660_at	acyl-CoA synthetase long-chain family member 3

7	*ACSL4*	202422_s_at	acyl-CoA synthetase long-chain family member 4

8	*ACSL5*	218322_s_at	acyl-CoA synthetase long-chain family member 5

9	*ADH1A*	207820_at	alcohol dehydrogenase 1B (class I), beta polypeptide; alcohol dehydrogenase 1A (class I), alpha polypeptide; alcohol dehydrogenase 1C (class I), gamma polypeptide

10	*ADH6*	207544_s_at	alcohol dehydrogenase 6 (class V)

11	*ADIPOQ*	207175_at	adiponectin, C1Q and collagen domain containing

12	*AGPAT2*	210678_s_at	1-acylglycerol-3-phosphate O-acyltransferase 2 (lysophosphatidic acid acyltransferase, beta)

13	*ANGPTL4*	221009_s_at	angiopoietin-like 4

14	*APOA4*	206894_at	apolipoprotein A-IV

15	*APOC3*	205820_s_at	apolipoprotein C-III

16	*AQP7*	206955_at	aquaporin 7

17	*ARSA*	204443_at	arylsulfatase A

18	*ASAH1*	210980_s_at	N-acylsphingosine amidohydrolase (acid ceramidase) 1

19	*CERK*	218421_at	ceramide kinase

20	*CETP*	206210_s_at	cholesteryl ester transfer protein, plasma

21	*CYP7A1*	207406_at	cytochrome P450, family 7, subfamily A, polypeptide 1

22	*DGAT1*	202344_at	diacylglycerol O-acyltransferase homolog 1 (mouse)

23	*EHHADH*	205222_at	enoyl-Coenzyme A, hydratase/3-hydroxyacyl Coenzyme A dehydrogenase

24	*FABP2*	207475_at	fatty acid binding protein 2, intestinal

25	*FUT2*	208505_s_at	fucosyltransferase 2 (secretor status included)

26	*FUT4*	209892_at	fucosyltransferase 4 (alpha (1,3) fucosyltransferase, myeloid-specific)

27	*FUT5*	210398_x_at	fucosyltransferase 5 (alpha (1,3) fucosyltransferase)

28	*FUT9*	207696_at	fucosyltransferase 9 (alpha (1,3) fucosyltransferase)

29	*GCDH*	203500_at	glutaryl-Coenzyme A dehydrogenase

30	*GK2*	215430_at	glycerol kinase 2

31	*GLA*	214430_at	galactosidase, alpha

32	*HADHA*	208629_s_at	hydroxyacyl-Coenzyme A dehydrogenase/3-ketoacyl-Coenzyme A thiolase/enoyl-Coenzyme A hydratase (trifunctional protein), alpha subunit

33	*LTA*	206975_at	lymphotoxin alpha (TNF superfamily, member 1)

34	*MTTP*	205675_at	microsomal triglyceride transfer protein

35	*NPC1L1*	220106_at	NPC1 (Niemann-Pick disease, type C1, gene)-like 1

36	*NR1H3*	203920_at	nuclear receptor subfamily 1, group H, member 3

37	*OLR1*	210004_at	oxidized low density lipoprotein (lectin-like) receptor 1

38	*PCK2*	202847_at	phosphoenolpyruvate carboxykinase 2 (mitochondrial)

39	*PLTP*	200661_at	phospholipid transfer protein

40	*PPARG*	208510_s_at	peroxisome proliferator-activated receptor gamma

41	*RXRB*	209148_at	retinoid × receptor, beta

42	*RXRG*	205954_at	retinoid × receptor, gamma

43	*SGMS1*	212989_at	sphingomyelin synthase 1

44	*ST8SIA1*	210073_at	ST8 alpha-N-acetyl-neuraminide alpha-2,8-sialyltransferase 1

45	*UCP1*	221384_at	uncoupling protein 1 (mitochondrial, proton carrier)

46	*UCP3*	207349_s_at	uncoupling protein 3 (mitochondrial, proton carrier)

47	*UGCG*	204881_s_at	UDP-glucose ceramide glucosyltransferase

### Replication of the results: meta-analyses

We also aimed to ensure that the results obtained from one microarray dataset were robust and could be replicated in other datasets. We queried the Oncomine database and retrieved microarray data from other relevant studies. We studied the association of gene expression with three outcomes--risk of breast cancer, risk of metastasis and risk of recurrence. We then combined these datasets meta-analytically using the random effects model of DerSimonian and Laird [13,14]. For these analyses the effect size was measured and expressed as the standardized mean difference (SMD) and its 95% confidence intervals. The Oncomine website reports the results as means, medians, quartiles and minimum and maximum values. Since the random-effects model assumes normal distribution of the effect measures, we first estimated the mean and standard error for each group (for example, for subjects with breast cancer; subjects with a metastatic event or subjects with recurring breast cancer) using the method described by Hozo et al. [15] We then estimated the SMDs. To depict the potential variability in the *HADHA *expression based on the probes used by individual studies, we conducted the meta-analyses separately for each combination of the study and the probe used. Each comparison represented a specific combination of the included study and the reporter used in the study. The between-study heterogeneity in this meta-analysis was examined using the I^2 ^statistic. Since expression data on all individual subjects was available for the outcome of risk of breast cancer, we also conducted individual patient data (IPD) meta-analysis [16]. For this we used the clustered unconditional logistic regression analyses [16,17] with disease status as a dichotomous dependent variable, comparison-specific z-scores as the predictor variable and comparison indicator as the clustering variable. Comparison-specific z-scores were estimated as the relative deviates (mean expression/standard deviation of expression) within each comparison group.

### Other statistical analysis

To quantify and test differential gene expression, we used two-tailed Student's t tests for unpaired samples. The clinical and statistical significance of the findings were presented as volcano plots. To account for multiple testing, we estimated the false discovery rates (q values) using the QVALITY software program [18]. Discriminant utility of each gene was assessed using non-parametric receiver operating characteristic (ROC) curve analysis. To group subjects based on their HADHA expression, we used a k-means clustering approach. All statistical analyses were conducted using Stata 10.0 software package (Stata Corp, College Station, Texas). We aimed for a type I error rate of 0.05 and a false discovery rate of 0.15.

## Results

### Differential expression analyses

**Figure 1:**
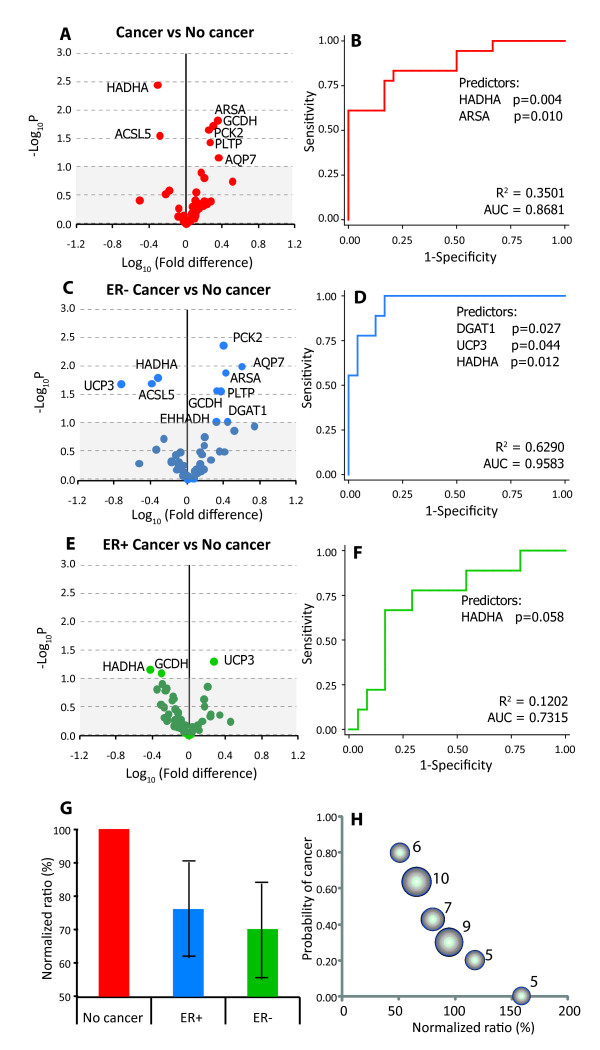
**Association of fatty acid and lipid metabolism related genes with the risk of breast cancer**. **(A-F) Association analyses**. Panels **A**, **C **and **E **show the volcano plots for cancer with no cancer, ER-versus no cancer and ER + versus no cancer comparisons, respectively. These plots depict the biological significance (log-fold change) on the X-axis and the statistical significance (-log P) on the Y-axis. Significance values above 0.1 are indicated by the grey shaded area in the volcano plots. Panels, **B**, **D **and **F **show the corresponding receiver-operating characteristic (ROC) curves for the final models from stepwise logistic regression analyses. The genes retained in the final model and their statistical significance is shown under the ROC curves, the variance explained by the model is shown as R^2 ^and the predictive accuracy is indicated by the area under the ROC curve (AUC). **(G) **Comparative expression of the *HADHA *gene in the indicated study groups. Error bars indicate 95% confidence intervals. **(H) **Bubble plot showing the dose-response relationship between *HADHA *expression and the risk of breast cancer. Each bubble represents one of the six clusters generated using k-means clustering algorithm based on the *HADHA *expression. The radius of the bubble is proportional to the number of subjects in that cluster (indicated by numbers next to the bubbles).

Using the shortlisted set of 47 genes shown in Table 1, we first determined if these genes were differentially expressed in subjects with cancer (n = 18) and those without (n = 24). The volcano plot (Figure 1A) showed that seven of the 47 genes were significantly differentially expressed between these study groups. These genes included five over-expressed genes (*AQP7, PLTP, PCK2, GCDH *and *ARSA*) and two under-expressed genes (*ACSL5 *and *HADHA*). Of these, *HADHA *was the most significant statistically. To account for the possible covariance among these gene expression values we conducted stepwise multivariate analyses using unconditional logistic regression and observed that only two genes--*HADHA and ARSA*--were retained in the final model (Figure 1B). This model explained 35% of inter-individual variability in breast cancer susceptibility with a predictive accuracy of 86.8%. Interestingly, when *HADHA *expression was removed from this model the *ARSA *lost its statistical significance but removal of *ARSA *did not affect the statistical significance of *HADHA*. This indicates that *HADHA *gene expression was the most important statistical predictor of altered risk of breast cancer.

### Does ER status influence the expression of *HADHA*?

To examine if this association could be influenced by the ER status, we conducted three sets of analyses. First, we studied whether *HADHA *expression was different based on the ER status. We found that the mean *HADHA *was not significantly differentially expressed by ER status (mean *HADHA *expression in subjects with ER + breast cancer = 6.00; in subjects with ER-breast cancer = 5.90; *p *= 0.462). Second, we adjusted the standard error estimates for the ER status using clustered logistic regression and observed that the statistical significance for the *HADHA *gene expression further increased (*p *= 0.0001) while that of the *ARSA *gene decreased (*p *= 0.082) indicating that the association of *HADHA *was unlikely to have been influenced by the ER status. Third, we constructed volcano plots and conducted stepwise logistic regression analyses by comparing the ER + and ER-subjects separately with subjects without cancer as the reference group. We observed (Figure 1C-F) that *HADHA *gene expression was the only consistent predictor across ER status but more so in the ER-subjects. Indeed, the q value for the *HADHA *gene was 0.15 for the cancer versus no cancer comparison, 0.13 for the ER-versus no cancer comparison but 0.88 for the ER + versus no cancer comparison. Two other genes (*UCP3 *and *DGAT1*) were retained in the final model of stepwise regression analyses when ER-subjects were compared to the no cancer group however this association was not observed when ER + subjects were compared to the same reference group.

### Graded risk of breast cancer based on *HADHA *expression

We next considered whether the association of *HADHA *gene expression with risk of breast cancer exhibited a threshold effect or whether it was a graded dose-response. For this, we used two approaches. First, we normalized the gene expression in the no cancer group to 100%. We found (Figure 1G) that the *HADHA *expression had fallen to 73% (95% CI 64%-83%) in subjects with cancer; with a higher expression in ER + subjects (76% of the no cancer group, 95% CI 61%-91%) than in ER-subjects (70% of the no cancer group, 95% CI 55%-85%). Second, the k-means clusters (which explained 95.9% of the variability in *HADHA *expression) clearly demonstrated a dose-response association (Figure 1H) such that more severe down-regulation of *HADHA *was associated with a greater risk of being in the breast cancer group.

### Meta-analyses of the differential expression of *HADHA*

Lastly, we examined the robustness of the differential expression of *HADHA *by conducting meta-analysis of published microarray studies comparing cases of breast cancer with subjects without breast cancer. Querying the Oncomine database, we found six studies [19-24] that represented 20 different comparisons of breast cancer patients with normal subjects (Table 2). The reasons for this larger number of comparisons were the different reporters used in the microarray experiments as well as the different subtypes of breast cancer reported by the studies.

**Table 2:** Comparisons included in the meta-analysis of differential *HADHA *expression

No	Author, Year	Ref	Controls	Cases	Breast cancer histology	Reporter
1	Zhao, 2004	[24]	3	37	Invasive ductal carcinoma	IMAGE:1473300

2	Zhao, 2004	[24]	3	21	Lobular carcinoma	IMAGE:1473300

3	Radvanyi, 2005	[21]	9	7	Invasive lobular carcinoma	BE297873

4	Radvanyi, 2005	[21]	9	32	Invasive ductal carcinoma	BE297873

5	Radvanyi, 2005	[21]	9	3	Invasive mixed carcinoma	BE297873

6	Radvanyi, 2005	[21]	9	3	Ductal carcinoma in situ	BE297873

7	Richardson, 2006	[22]	7	40	Ductal carcinoma	208629_s_at

8	Richardson, 2006	[22]	7	40	Ductal carcinoma	208630_at

9	Richardson, 2006	[22]	7	40	Ductal carcinoma	208631_s_at

10	Karnoub, 2007	[20]	15	7	Invasive ductal carcinoma	208629_s_at

11	Karnoub, 2007	[20]	15	7	Invasive ductal carcinoma	208630_at

12	Karnoub, 2007	[20]	15	7	Invasive ductal carcinoma	208631_s_at

13	Turashvili, 2007	[23]	20	5	Invasive ductal carcinoma	208629_s_at

14	Turashvili, 2007	[23]	20	5	Invasive ductal carcinoma	208629_s_at

15	Turashvili, 2007	[23]	20	5	Invasive ductal carcinoma	208630_at

16	Turashvili, 2007	[23]	20	5	Invasive lobular carcinoma	208630_at

17	Turashvili, 2007	[23]	20	5	Invasive lobular carcinoma	208631_s_at

18	Turashvili, 2007	[23]	20	5	Invasive lobular carcinoma	208631_s_at

19	Finak, 2008	[19]	6	53	Invasive breast carcinoma	A_24_P242688

20	Finak, 2008	[19]	6	53	Invasive breast carcinoma	A_24_P353964

**Figure 2:**
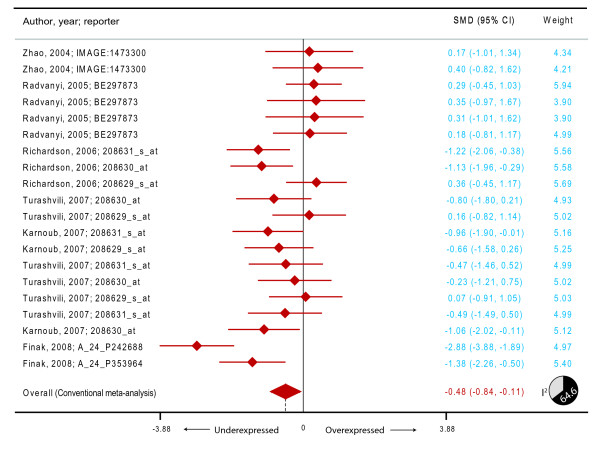
**Meta-analysis of the differential expression of the *HADHA *gene in breast cancer compared to subjects without breast cancer**. The figure shows a forest plot with filled diamonds indicating the point estimates and error bars indicating the 95% confidence interval around the standardized mean difference. The summary effect measure is shown as a filled diamond whose center (dashed vertical line) indicates the point estimate and the width indicates the 95% confidence interval.

We first observed that the mean expression levels for *HADHA *probes (expressed as log transformed values) were widely different across the six studies (Zhao et al. [24]:-0.33, Radvanyi et al. [21]: 3.08, Richardson et al. [22]: 5.29, Karnoub et al. [20]: 2.97, Turashvili et al. [23]: 3.92 and Finak et al. [19]:-2.65). We therefore transformed these values into comparison-specific z-scores (mean expression for a comparison/standard deviation of expression for that comparison). Upon this z-transformation, all the studies had a mean z-score of 0 and a standard deviation of 1. We conducted meta-analyses on the *HADHA *expression z-scores. Using the DerSimonian and Laird model, we observed (Figure 2) that the summary SMD (filled diamond in Figure 2) was-0.48 (95% CI-0.84--0.11). Considering the statistical properties of SMD it is possible to transform this into probability [25]. This transformation indicated that there was an average 18.4% reduction in expression of *HADHA *(95% CI 4.5%-30.0%) in cases of breast cancer as compared to normal subjects. Interestingly, this significant reduction in the expression of *HADHA *was observed in spite of the high degree of heterogeneity (I^2 ^64.6, *p *< 0.001, pie-chart in Figure 2) between the comparisons due to different cancer subtypes, reporters used in various studies and other study characteristics.

We observed that the invasive ductal carcinoma (*p *= 0.046) and unspecified invasive breast carcinoma (*p *= 0.005) showed a significant under-expression of *HADHA *gene but lobular carcinoma (*p *= 0.781), invasive lobular carcinoma (*p *= 0.780) or invasive mixed carcinoma (*p *= 0.717) did not show a significant alteration of *HADHA *gene expression. Alternatively, we conducted the IPD meta-analysis using logistic regression analyses. We found that the odds ratio for breast cancer was 0.74 (95% CI 0.60-0.92) after clustered analyses. Thus, there was a 26% reduction in the risk of breast cancer per unit increase in z-scores. These values show a striking resemblance with the findings observed in the Graham et al. dataset and demonstrate the replicability of our findings.

**Figure 3:**
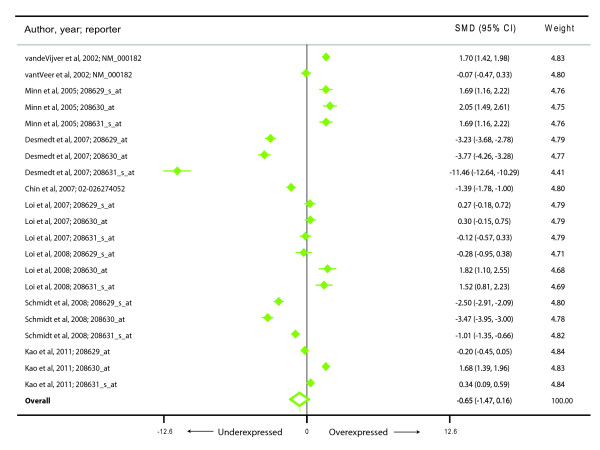
**Meta-analysis of the differential expression of the *HADHA *gene in breast cancer patients with and without metastatic events**. The figure shows a forest plot with filled diamonds indicating the point estimates and error bars indicating the 95% confidence interval around the standardized mean difference. The summary effect measure is shown as a hollow diamond whose center (dashed vertical line) indicates the point estimate and the width indicates the 95% confidence interval.

**Figure 4:**
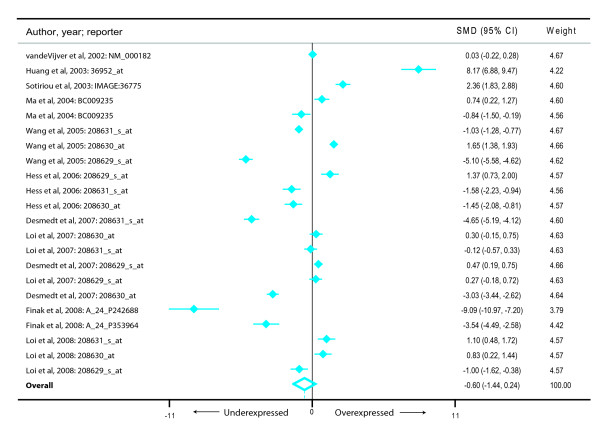
**Meta-analysis of the differential expression of the *HADHA *gene in breast cancer patients with and without recurrence events**. The figure shows a forest plot with filled diamonds indicating the point estimates and error bars indicating the 95% confidence interval around the standardized mean difference. The summary effect measure is shown as a hollow diamond whose center (dashed vertical line) indicates the point estimate and the width indicates the 95% confidence interval.

We also investigated if *HADHA *expression was associated with an altered risk of metastasis and recurrence. For risk of a metastatic event we found nine studies [26-33] representing 430 metastatic events and 1104 metastasis-free cancers (Figure 3). Subjects who developed a metastatic event during follow-up had a reduced *HADHA *expression (summary effect size-0.65, 95% CI-1.47-0.16) but this was not statistically significant (*p *= 0.117). Also, there was a very high degree of between-study heterogeneity (I^2 ^98.8%). Similarly, for the outcome of the risk of recurrence (Figure 4), we found that there were 10 studies [19,27,29,30,32,34-38] representing 484 recurring and 890 non-recurring breast cancers. Meta-analysis demonstrated that although there was a consistent decrease in average *HADHA *expression in patients with a recurring form of breast cancer (summary effect size-0.60, 95% CI-1.44-0.24), the finding was neither statistically significant (*p *= 0.160) nor homogeneous (I^2 ^= 98.7%) across studies.

## Discussion

Our analyses of the microarray dataset based on the Graham et al. [5] study demonstrated a consistent, strong and significant association of the *HADHA *gene expression in histologically normal epithelium with the likelihood of breast cancer. Moreover, this observation was further substantiated by the meta-analysis of other published studies. Only one study has previously reported differential association of this gene with regard to BRCA1 positive, BRCA2 positive and sporadic malignant tumors of the breast [39]. Our results further support the putative involvement of *HADHA *in breast cancer susceptibility.

### Biological plausibility

Biological significance of our novel observations should be considered in the light of the following facts. First, the *HADHA *gene (chromosomal location 2p23) codes for the four alpha chains in the 8-meric mitochondrial trifunctional protein (TFP) [40]. This enzyme performs three cardinal functions in the β-oxidation of long chain fatty acids by catalyzing the activities of the 2-enoyl-CoA hydratase (ECH), L-3-hydroxyacyl-CoA dehydrogenase (HACD) and 3-ketoacyl-CoA thiolase (KACT). Of these three, the first two enzymes (ECH and HACD) are specifically catalyzed by the alpha chains of TFP. Severe deficiency (< 50% of normal activity) of TFP is known to be associated with life-threatening manifestation of the long chain 3-hydroxyacyl-CoA dehydrogenase deficiency [41]. However, the effects of a milder deficiency of TFP (for example, activity between 50%-80% of the normal) are currently unknown. Our results indicate that breast cancer patients had 18-30% decreased expression of *HADHA *gene. We therefore hypothesize that there may be a compromised metabolism of long chain fatty acids in breast cancer due to a relative deficiency of the alpha chains of TFP. In this context, it is noteworthy that a recent large genome-wide association study [42] found a strong association of breast cancer with a polymorphism in the gene encoding enoyl CoA hydratase domain containing 1 (*ECHDC1*), which also partakes in the integrity of the TFP.

Second, the efficacy of β-oxidation of n-3 and n-6 long chain fatty acids can be tissue- and location- specific. For example, in rat livers it has been shown that the n-3/n-6 ratio influences peroxisomal but not mitochondrial β-oxidation [43]. In contrast, mitochondrial β-oxidation of long chain fatty acid has been implicated in breast cancer pathogenesis [42]. We also could not demonstrate a significant association of the genes involved in the PPAR-γ pathway reinforcing the possibility that mitochondrial rather than peroxisomal β-oxidation of long chain fatty acids may be more critical in breast carcinogenesis. Third, *HADHA *occupies an important position in the network of genes that have been implicated in autophagy and apoptosis [44]. Finally, triangulation of the following facts lends additional credence to our observations: i) intact epithelium of mammary glands has the ability to act as stem cells for carcinogenesis [2]; ii) n-3 long chain fatty acids have the ability to target such stem cells [45]; and iii) *HADHA *is involved in the mitochondrial β-oxidation of long chain fatty acids. Together these observations from published literature strongly support the biological plausibility of our finding that *HADHA *is differentially expressed in subjects with and without breast cancer.

### Limitations

Our study has all the limitations implicit in any microarray association study and meta-analyses. In addition, there are three more limitations. First, although there is a strong circumstantial evidence that favors an inference of *HADHA *expression-breast cancer association, it must be realized that robust functional studies are required before this association can be conclusively claimed. Our study does not have a component of functional assays that can help put these results in a biological perspective. Second, due to limitations imposed by the microarray platform used in the primary study, we could not evaluate the potential association of a large number of additional lipid and fat metabolism related genes with the risk of breast cancer. Inclusion of those genes may not only affect the q values associated with *HADHA *but also may provide a more comprehensive understanding of the role of fatty acids in breast cancer. Thirdly, although consistent, the observed differential expression of *HADHA *with cancer progression (as reflected by risk of metastasis and recurrence) is statistically inconclusive.

## Conclusions

Our study has three important implications--biological, methodological and epidemiologic. Biologically, our study has identified a novel target gene that corroborates the existing knowledge about the role of long chain fatty acids in breast cancer and provides interesting directions for further research in this area. Also, our findings put the focus on the putative functional aspects of mitochondria and TFP in breast carcinogenesis.

From a methodological standpoint, our study shows that high dimensionality of omics-type datasets is fraught with the vexing problem of finding strong associations at the cost of potentially missing weaker but biologically meaningful associations. Literature addressing the issue of multiple comparisons in large volume datasets focuses primarily on the possibility of finding false positive associations [46]. However, there exists a demonstrable probability that such high-volume datasets may also falsely mask true associations. It is likely that the Graham et al. study did not report a significant association of *HADHA *with the risk of breast cancer due to a large number of multiple comparisons. The fact that we discovered an association of *HADHA *with breast cancer shows that microarray dataset analysis (as well as analyses of other large datasets like genome-wide association studies, proteomics data or metabolomics datasets) may benefit by using targeted subset analyses based on functional annotation and conceptual understanding of the molecular mechanisms in disease. Finally, in an epidemiological context, our study shows that error in long chain fatty acid metabolism in the breast tissue might herald the onset of carcinogenesis and thus can be helpful for the primordial prevention of breast cancer.

## Abbreviations

ER: Estrogen receptor; n-3 PUFA: Omega--3 polyunsaturated fatty acids; n-6 PUFA: Omega--6 polyunsaturated fatty acids; SFA: Saturated fatty acids; SMD: Standardized mean difference; CI: Confidence interval.

## Competing interests

The authors declare that they have no competing interests.

## Authors' contributions

MM and HK conceptualized the study, collected data, conducted analyses and wrote the manuscript. Both authors have read and approve the manuscript.

## Supplementary Material

Additional file 1 **Table S1**. Excel table containing detailed annotation of the 136 genes related to fat and lipid metabolism that were primarily selected for analyses. Click here for file

Additional file 2 **Table S2**. Excel table containing detailed annotation of the 47 genes included in this study related to fat and lipid metabolism that were primarily selected for analyses. Click here for file
